# Signal-to-noise, spatial resolution and information capacity of coherent diffraction imaging

**DOI:** 10.1107/S2052252518010941

**Published:** 2018-09-15

**Authors:** Timur E. Gureyev, Alexander Kozlov, Yakov I. Nesterets, David M. Paganin, Andrew V. Martin, Harry M. Quiney

**Affiliations:** a ARC Centre of Excellence in Advanced Molecular Imaging, School of Physics, University of Melbourne, Parkville, Victoria 3010, Australia; b Faculty of Health Sciences, University of Sydney, Sydney NSW 2006, Australia; c School of Science and Technology, University of New England, Armidale NSW 2351, Australia; d Manufacturing, CSIRO, Clayton, Victoria 3168, Australia; e School of Physics and Astronomy, Monash University, Clayton, Victoria 3800, Australia; f School of Science, RMIT University, Melbourne, Victoria 3001, Australia

**Keywords:** coherent diffractive imaging, signal-to-noise ratio, spatial resolution, information capacity

## Abstract

It is shown that the average signal-to-noise ratio (SNR) in the three-dimensional electron-density distribution of a sample reconstructed by coherent diffractive imaging cannot exceed twice the square root of the ratio of the mean total number of scattered photons detected during the scan and the number of spatially resolved voxels in the reconstructed volume. This result leads to an upper bound on Shannon’s information capacity of this imaging method by specifying the maximum number of distinguishable density distributions within the reconstructed volume when the radiation dose delivered to the sample and the spatial resolution are both fixed. If the spatially averaged SNR in the reconstructed electron density is fixed instead, the radiation dose is shown to be proportional to the third or fourth power of the spatial resolution, depending on the sampling of the three-dimensional diffraction space and the scattering power of the sample.

## Abstract 2

Signal-to-noise ratio, spatial resolution and information capacity of tomographic coherent diffractive imaging are investigated; the results are expected to be useful for the design and analysis of synchrotron and XFEL-based diffractive imaging experiments.

## 1. Introduction   

The tomographic form of coherent diffraction imaging (CDI) is typically concerned with the reconstruction of the three-dimensional spatial distribution of electron density in a sample from a set of X-ray diffraction patterns collected in the far (Fraunhofer) region at different rotational positions of the sample (Sayre *et al.*, 1998 ▸; Robinson *et al.*, 2001 ▸; Marchesini *et al.*, 2003 ▸; Chapman *et al.*, 2006 ▸). The issue of sample damage, as a result of the X-ray dose delivered to the sample in the process of imaging, is central to this method, particularly when high-resolution imaging of biological samples is considered. It has been shown that when the signal-to-noise ratio (SNR) in the reconstructed electron-density distribution is fixed, the X-ray dose delivered to the sample is proportional to the third (Bergh *et al.*, 2008 ▸) or fourth (Howells *et al.*, 2009 ▸) power of the spatial resolution. For single non-crystalline biological samples, this limits the spatial resolution to approximately 10 nm (Howells *et al.*, 2009 ▸). More recently, it has been demonstrated that the use of ultra-short pulses (<100 fs) at X-ray free-electron laser (XFEL) sources allows one to ‘outrun’ the radiation damage by collecting many diffraction images from identical samples, each image being produced before the sample is destroyed by the X-ray pulse (Chapman *et al.*, 2011 ▸; Quiney & Nugent, 2011 ▸; Martin *et al.*, 2015 ▸). As a result, the dose limits established for biological samples with synchrotron sources (Howells *et al.*, 2009 ▸) do not apply for XFEL experiments (Chapman *et al.*, 2011 ▸). There is an active effort to push XFEL imaging of single biological particles to atomic resolution and compete with X-ray crystallography and cryo-electron microscopy (Miao *et al.*, 2015 ▸). Despite the extreme intensity of XFEL pulses, they scatter as few as 10^2^–10^3^ photons per protein and the measured diffraction images are extremely noisy. The SNR is improved when a large number of diffraction images are assembled into a three-dimensional set, and it is predicted that 10^5^–10^6^ diffraction patterns will be required for the three-dimensional imaging of a protein (Tegze & Bortel, 2012 ▸). However, a number of challenging problems exist in this form of CDI, such as finding the unknown orientation of each imaged sample from the low-SNR diffraction patterns and reconstructing the phase distribution from diffraction intensity measurements. The questions of signal, noise and spatial resolution are central to these problems (Elser, 2009 ▸; Kirian *et al.*, 2011 ▸).

In CDI, the three-dimensional diffraction intensity volume (produced by an orientation-determination algorithm in the case of XFEL-type CDI) serves as the input for a phasing algorithm which ultimately recovers the real-space electron distribution in the sample. The SNR of the three-dimensional diffracted intensity volume impacts the accuracy of the recovered image. The impact of noise on popular CDI phasing algorithms has been characterized (Williams *et al.*, 2007 ▸) and several innovations to improve robustness-to-noise have been proposed (Loh *et al.*, 2010 ▸; Dilanian *et al.*, 2010 ▸; Martin *et al.*, 2012 ▸). Since phase retrieval is generally a non-linear problem, the impact of noise has diverse effects ranging from reduced resolution through to the failure of phasing algorithms to converge in more challenging cases. In the present paper, we do not study the noise tolerance of orientation-determination or phasing algorithms, which has been considered elsewhere (Loh & Elser, 2009 ▸; Giannakis *et al.*, 2012 ▸; Williams *et al.*, 2007 ▸; Loh *et al.*, 2010 ▸; Dilanian *et al.*, 2010 ▸). Rather we consider SNR limits that can be reached assuming particle orientation and phases are known. In this sense, our results provide an upper limit for the maximum achievable SNR in the electron-density distribution in the sample reconstructed at a certain spatial resolution if the particle orientation and phases are accurately determined by suitable algorithms. The obtained results hold under the assumption that there is no prior information about the sample, which is often not the case.

The impact of noise in XFEL diffraction imaging has been previously studied for the determination of particle orientation (Loh & Elser, 2009 ▸; Giannakis *et al.*, 2012 ▸) and for phase retrieval (Williams *et al.*, 2007 ▸; Loh *et al.*, 2010 ▸; Dilanian *et al.*, 2010 ▸). In the case of orientation determination, Bayesian methods (Loh & Elser, 2009 ▸) and manifold-based methods (Giannakis *et al.*, 2012 ▸) have been developed to overcome the low SNR of individual images by analysing the data as an ensemble. Many ensemble approaches avoid assigning each measurement a specific orientation. Instead, they use probabilistic or geometric methods to merge the ensemble of two-dimensional tomographic projections into a three-dimensional diffraction intensity volume. A one-dimensional proof-of-principle demonstration of a Bayesian orientation algorithm was successful with only 2.5 photons per pattern on average (Philipp *et al.*, 2012 ▸) and there are information-theoretic arguments that the XFEL signals for individual proteins will be sufficient for orientation determination (Loh *et al.*, 2010 ▸). However, it is critical to accurately estimate the number of required diffraction patterns, which, in turn, determines the experimental requirements for sample preparation, delivery and data collection.

**Figure 1:**
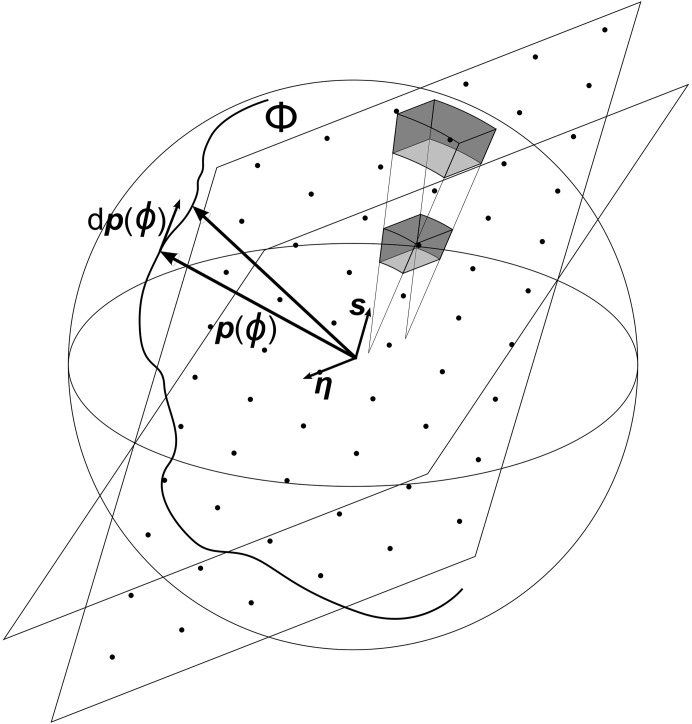
Sampling geometry of the diffraction space in a CDI experiment. The vector **p**(ϕ) is normal to the detector plane and extends from the centre of a fixed sphere, enclosing the sample to its surface with the angular coordinate *ϕ* parametrizing the curve Φ drawn by **p**(ϕ) on the surface of the sphere when the sample is rotated. The Cartesian coordinates (*s*, η) in the detector plane are fixed by the requirement that *s* is parallel to the vector *d* **p**(ϕ) tangential to Φ. Sampling volumes |Δ**q**|  = *s*Δ*s*ΔϕΔη around two different diffraction data points are indicated by greyscale shading (a more detailed description can be found in Section 4).

We investigate the limits for SNR and spatial resolution in three-dimensional CDI from the point of view of the noise–resolution uncertainty principle (Gureyev *et al.*, 2014 ▸, 2016 ▸). This principle states that, at a fixed radiation dose, the SNR and spatial resolution can almost always be traded for each other, but the square of their ratio, normalized by the incident photon fluence, is limited from above by the scattering power of the sample, *i.e.* the fraction of incident photons that are scattered by the sample (Gureyev *et al.*, 2016 ▸). ‘Naive’ considerations tell us that, when the noise in the collected CDI data is dominated by Poisson-distributed photon-shot noise and, therefore, the squared SNR in a detector pixel is proportional to the number of collected photons, the squared SNR in the three-dimensional tomographic data should in principle be proportional to the third power of the spatial resolution Δ*_r_* in the reconstructed sample. Indeed, when the linear dimension of a voxel in the sample is reduced twofold from Δ*_r_* to Δ*_r_*/2, its volume is reduced eightfold, from Δ*_r_* ^3^ to Δ*_r_* ^3^/8, and the number of photons scattered by the volume will generally also decrease by a factor of eight. It transpires that this ‘naive’ view is correct in the case of CDI data uniformly sampled in reciprocal space, in which case the squared SNR in the reconstructed sample is indeed proportional to Δ*_r_* ^3^. More precisely, as the SNR is a dimensionless quantity, its square is actually inversely proportional to the total number, *M*, of resolution voxels in the reconstructed sample, *M* = *V*/Δ*_r_* ^3^, where *V* is the reconstructed volume. In a real experiment however, when the sample is effectively rotated during a tomographic CDI scan and the corresponding image planes in the diffraction space rotate accordingly, the sampling is usually not uniform, as the distance between the data points on the periphery of the diffraction space is larger than the sampling distance in a close vicinity of the centre of rotation. In fact, the sampling distance generally increases in proportion to the radial variable in the cylindrical coordinates in reciprocal space. The fact that the corresponding sampling is closer to cylindrical than spherical will be discussed in detail later in the present paper. This spatially non-uniform sampling has a strong effect on the SNR in the reconstructed electron density. For a typical sample and planar illumination, the number of scattered photons would normally decrease as a function of the diffraction angle, and hence also as a function of the radial coordinate in reciprocal space. Therefore, the SNR in the collected CDI data, being equal to the square root of the number of registered photons, will also decrease as a function of the radial coordinate. On the other hand, the effect of non-uniform sampling density of the experimental data implies that, in the reconstruction process, the high-angle diffraction data has to be multiplied by a factor proportional to the radial variable of the cylindrical coordinates (Fig. 1). The combination of these two factors leads to the fact that, overall, the low-SNR diffraction data have higher weights in the reconstructed image compared with high-SNR data. In other words, the noise is amplified in the reconstruction more strongly relative to the useful signal. This effect is well known in computed tomography (CT), where the singular values of the inverse X-ray and Radon transforms increase in proportion to the square root of the radial order of the corresponding basis functions, making these inverse operators (moderately) unstable with respect to noise in the input data (Natterer, 1986 ▸). This noise amplification makes the squared SNR in the reconstructed data proportional to the fourth (rather than the third) power of spatial resolution, *i.e.* proportional to *M* ^−4/3^ = Δ*_r_* ^4^/*V* ^4/3^ (Howells *et al.*, 2009 ▸; Gureyev *et al.*, 2016 ▸).

As should be clear from the above considerations, the ‘fourth power law’: SNR^2^ ≃ *M* ^−4/3^ = Δ*_r_* ^4^/*V* ^4/3^ is likely to be dependent on two conditions. First, the sampling density in the diffraction space has to decrease in proportion to the radial variable in the cylindrical coordinates. As illustrated by the behaviour of the singular values in CT, this is a fundamental effect which cannot be overcome by a clever resampling of the registered data onto a uniform Cartesian grid, as part of the reconstruction process. Indeed, the corresponding interpolation of the data would inevitably increase the noise at least to the same degree as in an optimal reconstruction using the data on the original non-uniform sampling grid (Natterer, 1986 ▸). Second, in order for the fourth-power law to hold, the diffraction intensity must be a decreasing function of the radial coordinate. When this is not so, for example, for ‘sharply peaked’ samples that generate a near-flat distribution of diffraction signal in reciprocal space, the fourth-power law is replaced by the ‘third-power law’, SNR^2^ ≃ *M* ^−1^ = Δ*_r_* ^3^/*V*, the same as in the idealized case of spatially uniform sampling considered above. When the signal and noise have the same spatial distribution, it does not matter that the registered intensity data is multiplied by a factor proportional to the cylindrical radial coordinate in the reconstruction process, because this multiplication affects the signal and the noise in equal measure and the relative amplification of noise does not take place. These facts are studied in detail in Section 4 of the present paper, after the basic model of CDI image formation is considered in Section 2 and a corresponding generic expression for the SNR in the reconstructed electron density is derived in Section 3. Section 5 of the paper contains a brief investigation of the intrinsic imaging-quality characteristic (Gureyev *et al.*, 2014 ▸, 2016 ▸) and Shannon’s information capacity (Shannon, 1949 ▸; Cox & Sheppard, 1986 ▸) of CDI imaging systems. Appendix *A* [App appa] contains a mathematical derivation of the expectation value of the square root of a Poisson-distributed random variable, which is used to obtain the key result of Section 3. Appendix *B* [App appb] contains a list of the main symbols used in this paper.

## 2. Signal-to-noise ratio in diffracted intensity data   

Here we briefly outline some well known mathematical formulae describing image formation in CDI that are relevant to our analysis. Let
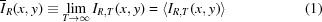
be the average intensity measured at point (*x*, *y*, *R*) in the image plane *z* = *R*, where 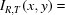 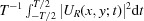 is the instantaneous light irradiance averaged over the exposure (or the illuminating pulse) time *T*, *U_R_*(*x*, *y*; *t*) is the complex wave amplitude (viewed as a wide-sense stationary stochastic process) in the image plane and the angular brackets denote the ensemble average. The variance of the intensity registered by a photon-counting detector with quantum efficiency κ is approximately equal to (Goodman, 1985 ▸; Mandel & Wolf, 1995 ▸; Gureyev, Nesterets *et al.*, 2017 ▸)
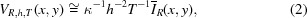
where *V*_*R*,*h*,*T*_(*x*, *y*) ≡  〈*I* ^2^_*R*,*h*,*T*_(*x*,*y*)〉  − 〈*I*_*R*,*h*,*T*_(*x*, *y*)〉^2^ is the variance of the intensity, *I*_*R*,*h*,*T*_(*x*, *y*), registered in a single effective detector pixel with linear size *h* (more precisely, *h* is the width of the point-spread function of the detector) and centered at the point (*x*, *y*, *R*). Equation (2) has been derived under the assumption that the average intensity 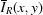 spatially varies slowly over distances comparable with *h*, and therefore is approximately invariant with respect to the convolution with the point-spread function of the detector.

In the semiclassical model of photodetection (Goodman, 1985 ▸; Mandel & Wolf, 1995 ▸), the quantity
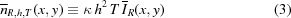
represents the mean number of photons detected over time *T* within the detector ‘pixel’ area *h* ^2^ centered at the point (*x*, *y*, *R*). Defining the squared SNR in the usual way as the ratio of the squared intensity to the intensity variance, 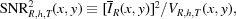 it is easy to verify from equations (1)–(3) that
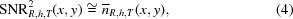
as expected in the case of Poisson photon counting statistics.

The above derivation of equation (4) assumes that the exposure time *T* is much larger than the coherence time *T*_c_ and the number of registered photons per pixel is not very large, so that 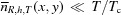 (Gureyev, Nesterets *et al.*, 2017 ▸), as is the case in a typical CDI experiment. If the latter condition is not satisfied, for instance, if the beam has a very high degree of temporal coherence, then the SNR may depend not only on the photon counting statistics inherent to the photodetection process, but also on the photon statistics intrinsic to the radiation source (‘self noise’) (Mandel & Wolf, 1995 ▸). Finally, equation (4) also ignores the effect of sample damage, which can lead to a variation of the scattering potential during the exposure time. This type of effect has been considered previously in a CDI context in a number of publications (Quiney & Nugent, 2011 ▸; Martin *et al.*, 2015 ▸).

Let us now describe how the SNR is related to the properties of the sample in the quasi-monochromatic case, within the constraints discussed in the previous paragraph. We assume that the incident beam is a plane wave propagating along the optic axis *z*, having spatially uniform intensity distribution, *I*(*x*, *y*, 0) = *I*_in_, and a flat phase, φ(*x*, *y* 0) = 0 in the object plane *z* = 0. We also assume that the scattering of the radiation by the sample is time-independent and weak, so that the standard first Born approximation can be applied to describe the scattering. Finally, we assume that the scattered beam is paraxial, allowing us to use the Fresnel diffraction integral for the propagated amplitude. Under these assumptions, the beam intensity in the far (Fraunhofer) field, *R* ≫ *A* ^2^/λ, where *A* is the diameter of the sample and λ is the mean wavelength of the radiation, can be described by the expression
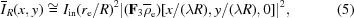
where 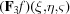 = 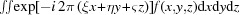 is the three-dimensional Fourier transform of *f*(*x*, *y*, *z*), *r*_e_ is the classical electron radius and 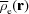 is the electron-density distribution in the sample (Cowley, 1995 ▸). As the primary unperturbed beam is typically blocked from the detector in this type of experiment, the diffracted intensity in the vicinity of the origin of coordinates may be unknown.

Substituting the expression for the diffracted intensity from equation (5) into equation (3), and then into equation (4), we obtain
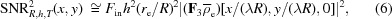
where *F*_in_ = κ*TI*_in_ is the uniform incident photon fluence (number of detected photons per unit area, which is assumed to be known) in the object plane, corresponding to the exposure time *T* and the detector efficiency κ. Using the terminology from the work by Gureyev *et al.* (2014 ▸, 2016 ▸), we can state that the two-dimensional ‘direct’ intrinsic imaging quality of the considered CDI setup is equal to
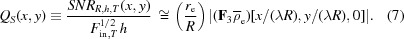
The quantity *Q_S_* characterizes the efficiency of utilization of incident photons by the imaging system in terms of achieving a certain SNR and spatial resolution (the latter two can be traded for each other at a fixed radiation dose delivered to the sample). However, as CDI imaging is mainly concerned with the quality of reconstruction of the unknown internal structure of a sample, rather than that of the diffraction images, it is more interesting in this case to investigate the behaviour of the SNR and spatial resolution in the reconstructed electron density as a function of the radiation dose and other essential parameters.

## 3. Signal-to-noise ratio and spatial resolution in reconstructed electron density   

According to equation (5), the diffracted intensity 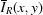 contains information about the distribution of the three-dimensional Fourier transform of the electron density, 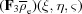 in a plane (ξ, η, 0) passing through the centre of the coordinates in reciprocal space. If the sample is rotated prior to exposure, equation (5) transforms into
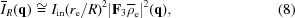
where 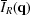 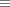 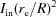 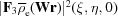 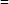 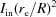 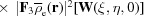 denotes the mean intensity distribution 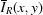 measured in the plane *z* = *R* after the sample has been rotated according to the orthogonal matrix **W**, **q** ≡ **W**(ξ, η, 0), ξ = *x*/(λ*R*) and η = *y*/(λ*R*). Here we used the fact that the rotation of the sample coordinate space is equivalent to the rotation of the reciprocal space owing to the linearity of the Fourier transform and the orthogonality of rotation matrices.

According to equation (8), if the diffracted intensity distributions are collected for a sufficiently broad range of rotational positions of the sample (the conditions that such a range must satisfy are discussed below), then the whole three-dimensional reciprocal space, except for a vicinity of the point (0, 0, 0), of the sample electron density can be probed. Furthermore, if the phase-retrieval problem can be solved for equation (8) in the sense that a unique complex amplitude, 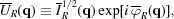 can be found on the basis of appropriate assumptions about the sample and the imaging setup, then the electron-density distribution in the sample can be obtained by three-dimensional inverse Fourier transform of the complex amplitude (Sayre *et al.*, 1998 ▸; Robinson *et al.*, 2001 ▸; Marchesini *et al.*, 2003 ▸; Chapman *et al.*, 2006 ▸),
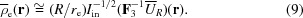
However, as the registered intensity is a stochastic distribution, then the reconstructed electron density is also going to be stochastic. In general, the random character of the measured diffracted intensity is determined by the properties of the X-ray source, the sample and the detector. We have assumed that the probabilistic properties of the source and the sample do not affect the random behaviour of the measured intensity, and the detector behaves as an ideal photon-counting detector. Consequently, we assume that the data measured at each pixel of the detector is a Poisson-distributed random variable *n*_*R*,*h*,*T*_(**q**) with the mean defined in equation (3). This random variable corresponds to the number of photons registered over the exposure time *T* by a photon-counting detector located on the optic axis at distance *R* from the sample, having quantum efficiency *κ* and effective pixel area *h* ^2^, with 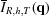 corresponding to the local radiation flux incident on the detector pixel during the exposure time. The electron-density distribution reconstructed from this random registered photon fluence data also represents a random variable, 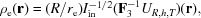 where the complex amplitude, *U*_*R*,*h*,*T*_(**q**), is obtained from the measured intensity *I*_*R*,*h*,*T*_(**q**) ≡ (κ*h* ^2^ *T*)^−1^ *n*_*R*,*h*,*T*_(**q**) with the help of a suitable phase-retrieval procedure.

We would like to determine the SNR and the spatial resolution in the reconstructed electron-density distribution ρ_e_(**r**), corresponding to SNR described by equation (4) and spatial resolution *h* in the collected diffraction intensity data. We estimate the following spatial average form of the squared SNR (Gureyev, Kozlov *et al.*, 2017 ▸) in the reconstructed density distribution,
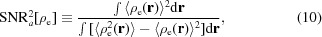
where angular brackets denote the ensemble average.

First, note that according to Parseval’s theorem and equation (8), we have 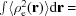 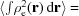 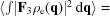 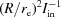 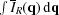 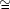 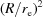 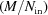 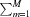 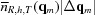, where 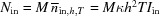 is the total number of incident photons irradiating the sample volume during the scan, *M* is equal to the total number of the detector pixels multiplied by the number of projections in the scan and the integral of the mean intensity distribution over the scanned area of reciprocal space has been approximated by a sum over discrete voxels with volume |Δ**q**_*m*_| centered at the measurement points **q**_*m*_. Estimation of the quantity 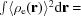 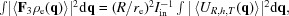 appearing in the numerator and denominator of equation (10), is more complicated technically than the evaluation of the integral 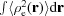 above. The corresponding mathematical details can be found in Appendix[App appa] *A*. Applying equation (24) from Appendix[App appa] *A* to equation (10), we obtain
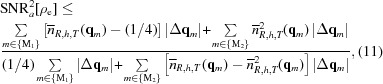
where *M*_1_ is the number of voxels in the subset {*M*_1_} corresponding to all data points in which the mean number of registered photons per pixel is larger than or equal to 1/2, and {*M*_2_} is the complementary subset corresponding to *M*_2_ voxels, where the mean number of registered photons per pixel is smaller than 1/2.

Consider first the case where the mean number of registered photons per pixel is smaller than 1/2 in almost all pixels, so that *M*_1_ ≪ *M*_2_ ≅ *M*. Then, because 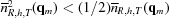 when 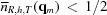, and 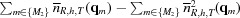 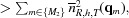 it follows from equation (11) that SNR^2^_*a*_[ρ_e_] < 1. This is clearly an undesirable case, as one would normally want to achieve a reconstruction of the electron-density distribution with an average SNR of at least 5, according to Rose criterion (Rose, 1948 ▸). If, on the other hand, the vast majority of pixels contain 1/2 or more photons on average, corresponding to *M*_2_ ≪ *M*_1_ ≅ *M*, then we obtain from equation (11) that 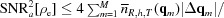 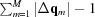, where the right-hand side is always larger than unity. This result represents a ‘tight’ upper bound for SNR^2^_*a*_[ρ_e_] in CDI in the sense that
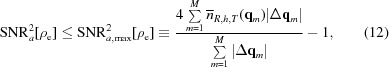
where the maximum value defined by the right-hand side of equation (12) can be reached for certain classes of diffracted intensities. In particular, this maximum is reached when 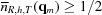 for all values of *m* and the reconstructed phase distribution is the same for all members of the ensemble of measured CDI scans (and hence the phase does not contribute to the variance of the complex amplitude) (see Appendix[App appa] *A*).

## 4. Effects of the scanning geometry and sample scattering strength on the SNR   

In the case of uniform voxel size in reciprocal space, for example, sampling on a regular Cartesian grid, we have |Δ**q**_*m*_|  =  |Δ**q**|  = *V* ^−1^ = (*M*Δ_*r*_^3^)^−1^, where Δ_*r*_ ≡ (*V*/*M*)^1/3^ is the corresponding spatial resolution in the object space. Therefore, 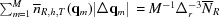, and equation (12) becomes 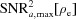 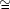 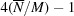, where 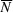 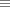 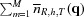 is the mean total number of registered photons in the diffraction images collected at all diffraction angles in one complete three-dimensional scan of the sample. Note that 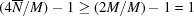, so that the SNR here is always larger than or equal to unity. Furthermore, when 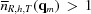 for all *m*, then 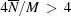, and it could be possible to approximate equation (13) further as
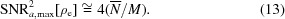
We will use this slightly less precise approximation below.

The fact that the squared SNR in equation (13) is proportional to the third power of the spatial resolution Δ_*r*_ ≡ (*V*/*M*)^1/3^, where *V* ≡  |Δ**q**|^−1^ is the reconstructed volume in the object space, rather than to the fourth power, as shown previously (Howells *et al.*, 2009 ▸), is the consequence of our (unrealistic) assumption that the voxel volume, |Δ**q**_*m*_|, is uniform throughout the diffraction space. This would be the case if the diffraction intensity data were collected on a regular Cartesian grid 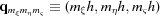 in the diffraction space, where *m_ξ_, m_η_* and *m_ς_* are integers, each spanning the set 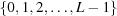, *L* ^3^ = *M*. However, this is difficult to achieve in a real CDI experiment. When the diffraction data are collected on a regular cylindrical grid, which is the case when the sample is rotated around a fixed axis, similar to conventional parallel-beam computed tomography (CT), the average squared SNR is known to be proportional to the fourth power of the spatial resolution (Natterer, 1986 ▸), and is given by
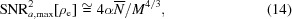
where *α* is a dimensionless constant of the order of unity, which depends on the sampling and interpolation schemes, but not on the collected diffraction intensities (numbers of photons) (Gureyev *et al.*, 2016 ▸). Compared with equation (13), here the additional power of Δ*_r_* in the expression Δ_*r*_^4^ = (*V*/*M*)^4/3^, appears as a consequence of the ‘ramp’ filtering, arising from the multiplication of the collected diffraction intensity data 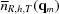 by coefficients proportional to *s* = (ξ^2^ + ς^2^)^1/2^ in the reconstruction process (Gureyev *et al.*, 2016 ▸). In turn, the presence of the ramp filter in the reconstruction process is a direct consequence of the linear non-uniformity of the elemental voxel volume as a function of the radial coordinate in the cylindrical case. Indeed, the elemental volume in cylindrical coordinates is equal to |d**q**| = *s*d*s*dηdϕ, assuming that the sample is rotated around the *y* axis (which is parallel to the *η* axis) during the scan (Fig. 1). This means that the higher order diffraction orders are actually being measured with lower precision (higher noise) when the detector pixels are uniform in size, because the sampling density linearly decreases as a function of the radial coordinate, *s*. In terms of the singular value decomposition (SVD) of the relevant reconstruction operator (which implements the inverse parallel-beam X-ray transform in three dimensions), the same effect leads to the singular values being equal to 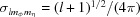, *s* = *lh*, which increase in proportion to the square root of the radial order *l* of the corresponding basis functions (Natterer, 1986 ▸). Such behaviour of the SVD usually results in the amplification of noise relative to the useful signal in the collected intensity data, and hence in lower SNR in the reconstructed sample, as in equation (14), compared with equation (13). This happens because the magnitude of a typical diffraction signal decreases as a function of |**q**|, and hence the relative amount of shot noise increases with |**q**|. Therefore, when the reconstruction amplifies high-order diffraction components, relative to the low-order ones, it effectively amplifies the noise with respect to the signal. This effect is demonstrated explicitly below.

Considering the case where the imaged sample is rotated in an arbitrary continuous way in three-dimensions, we note first that in order to provide a unique reconstruction of the sample, the set of all sample orientations used during the scan must satisfy the Orlov condition (Natterer, 1986 ▸; Orlov, 1975 ▸; Defrise *et al.*, 1993 ▸). It is easier to describe this condition by specifying that the sample is stationary, while the source and the detector are synchronously rotated around the sample (as *e.g.* in medical CT), instead of the equivalent real situation where the source and the detector are fixed and the sample is rotated (as in synchrotron-based CDI). Let **S** ^2^ be a fixed sphere enclosing the sample, and **p** denote a vector extending from the centre of the sphere to its surface and orthogonal to the detector plane. In the continuous case, the Orlov condition states that the line Φ, drawn on **S** ^2^ by the vector **p** while the detector is rotated during the scan, must intersect all great (equatorial) circles on **S** ^2^ (Fig. 1). For example, when the detector is rotated by 180° around the *y* (*η*) axis, as in conventional parallel-beam CT, the line Φ simply coincides with an equatorial semicircle. The Orlov condition is obviously satisfied in this case, because all equatorial circles on a sphere intersect any fixed equatorial semicircle. In general, when the detector is rotated arbitrarily around **S** ^2^, the line Φ does not lie in a single two-dimensional plane. However, it is still possible to introduce curved coordinates in a three-dimensional solid sphere enclosed by **S** ^2^, where the ‘rotation angle’ parameter *φ* parameterizes the line Φ on the surface, while two other coordinates (*s*, *η*) determine a position within the plane Π(ϕ) through the centre of the sphere with the normal vector pointing to Φ(*ϕ*) (Fig. 1). If Orlov’s condition is satisfied, the plane, Π(ϕ), sweeps the whole solid sphere, while the normal vector travels along the line Φ. If *s* is parallel to the vector d**p**(ϕ) tangential to line Φ at point *ϕ*, and *η* is perpendicular to *s* within the two-dimensional plane Π(ϕ), then the elemental three-dimensional volume in these coordinates will be the same as in the cylindrical case, *i.e.* |d**q**|  =  *s*d*s*dηdϕ. Therefore, in the case of optimal Shannon sampling, the SNR here will be the same as in the cylindrical case above, *i.e.* as in equation (14). Finally, if the rotational positions during the ‘scan’ are discrete (and can be random in the XFEL-type CDI), a sufficient condition for Shannon sampling can be formulated in the following way. Mapping all rotational positions Φ*_m_* of the sample as points on **S** ^2^, it should be possible to draw a continuous line Φ through all or a subset of the points Φ*_m_* in such a way that: (i) the line Φ satisfies the Orlov condition; (ii) the distance between points Φ*_m_* along the line Φ does not exceed *R*Δϕ = 2*R*/*L*, where *L* ^2^ is the total number of pixels in the detector (assuming that detector pixels are square and do not have gaps between them). The condition Δϕ = 2/*L* is further discussed below.

One might think that the difference between equations (13) and (14) is artificial, as any diffraction data collected on a cylindrical, spherical or other sufficiently dense and regular grid can be readily interpolated onto a suitable Cartesian grid, which would then lead to equation (13), rather than equation (14). However, it is known that the corresponding interpolation tends to amplify the noise in the measured data (Natterer, 1986 ▸), thus leading to a higher power of *M* in the denominator of equation (14). This is also true for random orientations of the sample, where the corresponding normal vectors **p** uniformly sample the whole sphere **S** ^2^. According to the Orlov condition, such sampling may be overdetermined (*e.g.* a sufficiently dense set of points on a single equatorial semicircle already provides optimal sampling). Therefore, the behaviour of the SNR will be expected to depend on the interpolation scheme employed for remapping the sampling points from the original scan grid points to a regular grid used in the reconstruction. Some methods may actually skip this remapping step and utilize the original grid points directly (Elser, 2009 ▸), but the stability of the method, and hence the SNR in the reconstructed electron-density distribution, will still inevitably depend on the geometry of the sampling grid, as demonstrated on a fundamental level by the behaviour of the singular values of the X-ray transform (Natterer, 1986 ▸). An estimate of the number of CDI projections required to provide Shannon’s sampling, in the case of random orientations of the sample uniformly distributed on a sphere, was given in Ekeberg *et al.* (2015 ▸). This number is consistent with the Orlov condition and the optimal angular sampling requirement in CT (Natterer, 1986 ▸), as discussed below.

Let us make the above arguments more precise by explicitly calculating the SNR in the case of cylindrical sampling of reciprocal space. Here we have 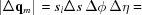 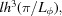 where *s*_*l*_ = (*l* − 1)*h*, Δ*s* = Δη = *h*, 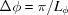, 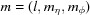, 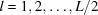, 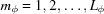, 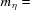 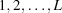, 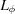 is the number of rotational positions (*i.e.* the number of projections) in the scan, *L* ^2^ is the number of pixels in the detector, so that 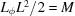 is the total number of data points in the scan. It is known (Natterer, 1986 ▸) that the optimal (Shannon) sampling is achieved when 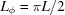. The corresponding number of diffraction patterns, (π/2)*L*, also agrees well with the estimate given for the XFEL-type CDI case in the work by Ekeberg *et al.* (2015 ▸). Assuming optimal sampling conditions, we have Δϕ = 2/*L*, *M* = (π/4)*L* ^3^ ≅ *L* ^3^ and |Δ**q**_*m*_|  = 2(*l*/*L*)*h* ^3^. As a consequence, 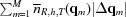 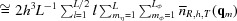 = 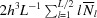, where 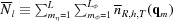 is the mean number of photons collected in the cylindrical shell with radius *s*_*l*_. Similarly, 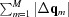 = 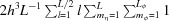 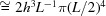, when *L*/2 ≫ 1. Substituting these expressions into equation (12), we find that, in the case of cylindrical sampling of the diffraction space, the maximum SNR achievable in CDI under conditions defined at the end of Section 3, when 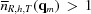 and the reconstructed phase is the same or all members of the ensemble of intensity measurements, is equal to
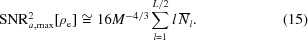
Let us consider two extreme cases.

(i) ‘Sharply peaked samples’ with a spatial distribution of electron density consisting of one or several very narrow peaks. For such samples, the distribution of diffracted intensity is almost flat (spatially uniform), 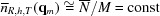, 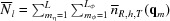 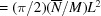 and 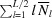 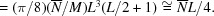 Substituting this into equation (15), we see that 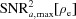 is described by equation (13), which was obtained earlier for arbitrary samples and uniform Cartesian sampling of the diffraction data. In particular, here SNR^2^_*a*,max_[ρ_e_] is also proportional to the third power of the spatial resolution, Δ_*r*_^3^ = *V*/*M*. The cylindrical non-uniform sampling does not reduce SNR in this case compared with the uniform Cartesian sampling, because the ramp filter, involving multiplication by *l* in the discrete form, affects the noise and the signal equally.

(ii) ‘Spatially flat’ samples with ‘slowly varying’ electron-density distributions which produce sharply peaked distributions of diffracted intensity. Let us assume that the dominant contribution to the total mean number of registered photons is provided by the first radial order, such that 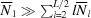. In this case, we have 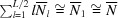. Substituting this into equation (15), we arrive at 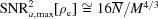, in agreement with equation (14) with α = 4. Cylindrical non-uniform sampling does decrease SNR in this case, because the ramp filter amplifies the noise in the higher diffraction orders much more strongly than the signal which predominantly comes from the first diffraction order.

It is logical to conclude that any real sample will belong somewhere in the range between the extremely sharp sample case (i) and the extremely flat sample case (ii) and, as a consequence, the squared SNR in the electron-density distribution reconstructed from CDI data collected from such a sample will satisfy the inequality 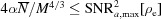 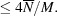


Note that the above results have been obtained with respect to the spatially averaged signal and noise variance in the reconstructed data. However, as for a typical sample, much fewer photons are scattered to high diffraction angles compared to small angles, the SNR in the high-order spatial frequencies of the reconstructed electron density is likely to be much lower compared with the SNR in the low-order spatial frequencies (except for very low-order frequencies, for which the diffraction data may not be registered at all, as it is masked by the unscattered transmitted beam and is blocked from the detector in a typical CDI experiment). In order to evaluate a true spatial resolution in the reconstructed sample, it may be necessary to estimate the SNR in the reconstructed high-order spatial frequencies. A relevant estimate can be readily obtained similarly to equation (12), in view of the one-to-one correspondence between the diffraction-intensity data collected at a particular **q** value and the spatial frequency of the same order in the reconstructed electron density according to equation (8). One only needs to replace the mean number of photons registered during the scan in all pixels by the corresponding mean number of photons registered in the pixels within the required range of **q** values. In particular, we can estimate the SNR in Fourier harmonics of radial order *l* of the reconstructed electron density. The following relationship can be derived similarly to equation (15):
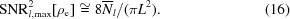
Consider, for example, the case of rapidly decreasing diffraction intensity, 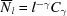, where 2 < γ < ∞ and *C*_γ_ is a constant. The total mean number of photons is equal to 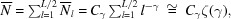 when *L*/2 ≫ 1, where ζ(γ) is the Riemann zeta function. This allows us to approximate 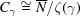. Then at *l* = *L*/2 we obtain 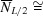 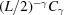 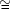 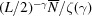 and 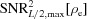 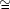 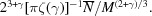 In a model case with *γ* = 4, this gives 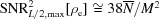. The last equation indicates that in order to maintain a fixed SNR in the highest radial order of spatial frequencies of the reconstructed electron density, it is necessary in this case to increase the total number of registered scattered photons in proportion to the sixth power of the spatial resolution, Δ_*r*_^6^ = (*V*/*M*)^2^. This is a very demanding requirement indeed, making the high-resolution CDI imaging rather challenging in this case and calling for the use of high-intensity X-ray sources, such as XFELs.

At the other extreme, when the diffraction intensity is flat, 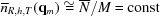, we have previously calculated that 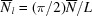 for any *l*. Substituting this into equation (16) with *l* = *L*/2, we obtain 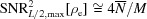. This result coincides with equation (13) as expected, because, in the case of a flat intensity, the SNR of any spatial Fourier frequency coincides with the average SNR. Consequently, in this case, in order to maintain a fixed SNR in the highest reconstructed Fourier frequencies, the number of scattered photons 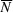 should be proportional to the third power of spatial resolution, Δ_*r*_^3^ = *V*/*M*.

## 5. Imaging quality characteristic and information capacity of CDI   

Let us consider further the case corresponding to equation (13), where SNR reaches the maximum possible value for CDI at a given radiation dose (which corresponds to the mean total number of diffracted photons 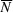) and a given spatial resolution [which corresponds to the total number of voxels *M*, with the spatial resolution Δ_*r*_ ≡ (*V*/*M*)^1/3^]. The three-dimensional reconstructive imaging quality characteristic of such a CDI system is equal to
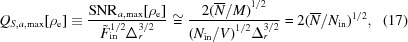
where *F*~_in_ = *N*_in_/*V* is the uniform three-dimensional incident photon fluence (number of photons per unit volume in the sample space, accumulated during the whole three-dimensional scan). The quantity 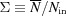 represents the scattering power of the imaged sample. Equation (17) is a remarkable result, given that it has been previously proven that for intensity-linear imaging systems the (two-dimensional, direct) imaging quality characteristic cannot exceed Σ^1/2^ (Gureyev *et al.*, 2016 ▸). This is also the case in equation (7) above. The extra factor of 2 in equation (17) can be traced back to the factor of 4 in the numerator of equation (12), which in turn appeared as a consequence of the statistics of the denominator of equation (10) in the most favourable case, *i.e.* when the noise in the collected CDI data had Poisson statistics, the mean number of photons in each pixel was larger than or equal to 1/2, and the reconstructed phase distribution in each two-dimensional diffraction image was the same for all members of the statistical ensemble. Ultimately, this effect is the consequence of the quadratic dependence of the registered diffraction intensities on the electron density in the sample, as described by equation (5). When the registered intensity is Poisson-distributed, the reconstructed electron density behaves as a square root of a Poisson-distributed random variable, and hence the ratio of the squared mean electron density to its variance is approximately equal to 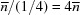, when 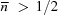, as shown in Appendix[App appa] *A*. For comparison, when measured intensities linearly depend on the electron density, as in the model case considered by Gureyev *et al.* (2016 ▸), the ratio of the squared mean to the variance is equal to 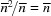, which is four times smaller than in the ‘quadratic case’. Note however, that the actual value of the imaging quality characteristic in equation (17) is always small, as 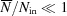 in accordance with the validity conditions of the first Born approximation used in the derivation of equation (17).

In cases where the SNR is proportional to the fourth power of the spatial resolution, as in equation (14), the imaging quality characteristic is equal to
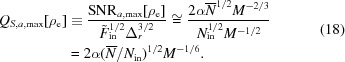
Therefore, in this case, the imaging quality characteristic decreases as a function of the number of resolution voxels. This is a direct consequence of the relative amplification of noise with respect to the useful signal in the case when both the sampling density and the number of scattered photons are decreasing as a function of the radial coordinate in reciprocal space.

The Shannon information capacity of an imaging system with *M* voxels and average SNR 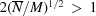, is equal to
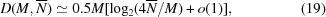
where the term *o*(1) is much smaller than unity when the ratio 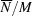 is large (Shannon, 1949 ▸; Cox & Sheppard, 1986 ▸; Gureyev *et al.*, 2016 ▸). This result indicates that a CDI system with *M* pixels, each one collecting more than one photon on average and 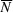 photons in total across all the pixels used in a scan, is capable of distinguishing between approximately 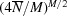 different samples, where each sample is represented by its electron-density values in *M* distinct voxels of equal size. In the case of communication systems, the same number represents the total of different messages that can be encoded by a system with a given number of channels and a given SNR (Shannon, 1949 ▸). Using the analogy with communication systems for the case of CDI, we state that the average SNR, 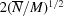 is approximately equal to the number of different levels of electron density that can be distinguished at each one of *M* voxels in the reconstructed sample, in the presence of Poisson photon shot noise in the registered diffraction intensities. Thus, we have a simple description of the maximal set of distinct objects that can be reconstructed from the data collected by a CDI system using 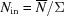 incident photons: each such object is represented by one of 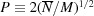 possible electron-density levels in each of *M* independent ‘resolution voxels’ within volume *V* in the object space.

## 6. Conclusions   

We have derived simple approximate expressions for the average SNR, intrinsic imaging quality and Shannon’s information capacity of CDI systems in terms of the total number of scattered photons collected in a three-dimensional scan and the number of resolution voxels in the reconstructed volume. We found that, at a fixed radiation dose delivered to the sample, the SNR may be proportional to the third or fourth power of the spatial resolution, depending primarily on the scattering characteristics of the sample. These results can help to estimate the upper bounds on the SNR of the electron density that can be achieved in CDI experiments if the phases and particle orientation are accurately determined. Such bounds are expected to be useful for the design of future XFEL experiments, especially when they are pushed to smaller particles or single protein molecules that scatter very weakly.

In the present study, we have only taken into account the noise in the CDI data which appears as a result of photon counting statistics in an ideal detector. Critical factors contributing to noise in experimental CDI data, such as sample damage during the exposure, fluctuations of the incident radiation between different exposures and variability in the sample configurations, have not been taken into account here. Discussions of the role of these factors in CDI experiments can be found in the previously cited publications (see *e.g.* Chapman *et al.*, 2011 ▸; Quiney & Nugent, 2011 ▸; Martin *et al.*, 2015 ▸; Miao *et al.*, 2015 ▸). However, a further analysis of the effect of these factors on SNR and spatial resolution in the reconstructed samples, using the approach taken in the present paper, may still be of interest. We believe that such analysis could potentially provide some new insights into these important problems and contribute to simplified guidelines for the planning of future XFEL experiments. We plan to obtain and present the relevant results in a related subsequent publication.

## Figures and Tables

**Figure 2:**
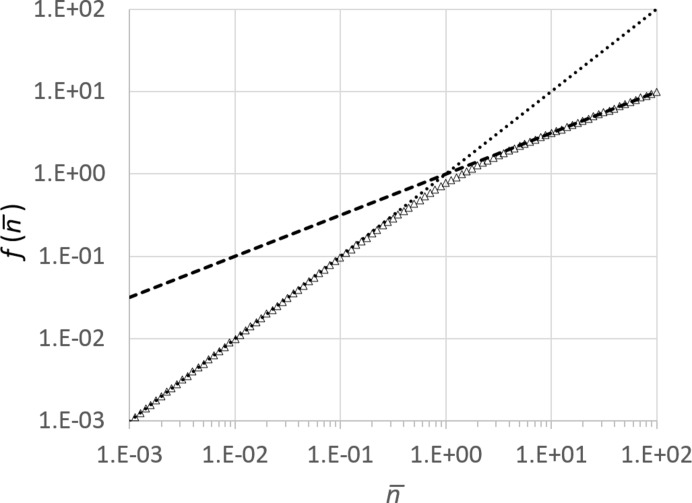
Numerically calculated values of 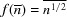 as a function of 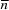 (triangles), and the corresponding asymptotes, 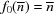 (dotted line) and 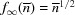 (dashed line), all plotted on a log–log scale.

**Figure 3:**
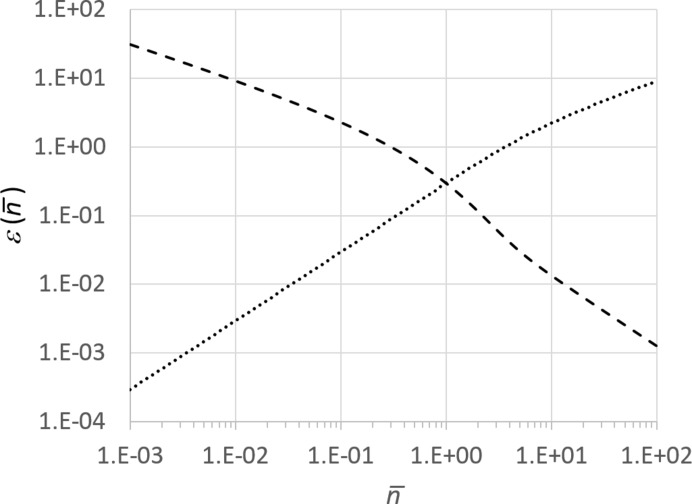
Numerically calculated values of the relative errors 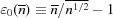 (dotted line) and 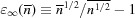 (dashed line) as a function of 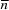, plotted on a log–log scale.

## Appendix A Expectation value of the square root of Poisson-distributed variable

In this Appendix we derive a sharp upper bound for the expression in the numerator of equation (10).

First, we consider the expectation value of the square root of the Poisson distribution,  , and find an approximation for its square value,  at any .

Following the suggestions at Mathematics *StackExchange* (2018 ▸), we can derive analytical approximations for the expectation value

when  and . In the first case, we expand the function *g*(*x*) = *x* ^1/2^ into the Taylor series around the point         , when . Substituting this into equation (20), we obtain

and hence , when .

When , we can approximate , when , and substitute this into the expression for *p*(*x*) in equation (20),

Therefore,

and hence , when .

Let us now estimate the error between the asymptotic approximations equations (21) and (22) and the exact value of  for different values of . Fig. 2 ▸ shows a comparison between the numerically calculated values of , as a function of , *i.e.* the function , and the corresponding asymptotes,  and .

It is easy to see from Fig. 2 that the two asymptotes approximate the function  quite well, not just when  and , but also when  and , respectively. In order to estimate the accuracy of these approximations, we calculated numerically the values of the function  in the range 0.001 ≤ *n* ≤ 100, and then evaluated the relative errors  and  within the same range. The graphs of these error functions are shown in Fig. 3.

The numerical estimates also show that the difference between the approximation

and the exact function , does not exceed 0.17, while the relative error does not exceed 0.35, for any .

Now consider the square modulus of the ensemble average of a complex random variable ,  = . We can apply the Cauchy–Schwarz inequality with the factors  |*U*_*j*_|^1/2^ and  to the last expression and obtain   Using this inequality, we then arrive at the following result,       It is easy to see that if the phases φ_*j*_ are the same for all *j*, then |〈*U*〉|^2^ = 〈|*U*|〉^2^; in this case the last inequality for the integral of the square of the mean electron density also becomes an equality.

We can now use the approximation from equation (23) for the function  to obtain

where *M*_1_ is the number of elements in the subset {M_1_} consisting of all data points from one scan in which the mean number of registered photons per pixel is larger than or equal to 1/2, and {M_2_} represents the complementary subset consisting of *M*_2_ data points where the mean number of registered photons per pixel is smaller than 1/2.

## Appendix B List of the main symbols used in the paper

*A*, diameter of the sample.

**F**_3_ *f*, three-dimensional Fourier transform of *f*.

*F*_in_, uniform incident photon fluence (number of detected photons per unit area).

*F*~_in_, uniform three-dimensional incident photon fluence (number of photons per unit volume).

*h*, detector pixel size.

*I*_in_, spatially uniform incident intensity.

, average intensity in the image plane *z* = *R*.

*I*_*R*,*h*,*T*_, intensity measured over time *T* in a pixel of size *h* located in the plane *z* = *R*.

*L*, square root of the total number of pixels in the detector.

, number of rotational positions (*i.e.* the number of projections) in the scan.

*M*, total number of resolution voxels in the reconstructed sample.

*n*_*R*,*h*,*T*_, number of photons detected over time *T* within area *h* ^2^ in the plane *z* = *R*.

*N*, total number of photons collected at all diffraction angles in one complete three-dimensional scan.

**p**, vector normal to the detector plane.

**q** = (ξ, η, ς), Cartesian coordinates in the diffraction space.

**q** = (*s*, η, ϕ), cylindrical coordinates in the diffraction space.

*r*_e_, classical electron radius.

**r** = (*x*, *y*, *z*), Cartesian coordinates in the object space.

*R*, distance between the sample and the detector.

*s* = (ξ^2^ + ς^2^)^1/2^, cylindrical radial coordinate in the diffraction space.

SNR_*a*_[ρ_e_], spatially averaged signal-to-noise ratio in the reconstructed electron density.

*T*, exposure time.

, complex wave amplitude.

*V*_*R*,*h*,*T*_, variance of *I*_*R*,*h*,*T*_.

*V*, reconstructed volume.

Δ*_r_*, spatial resolution in the reconstructed sample.

Δ**q**_*m*_, discrete voxel centered at the measurement point with index *m*.

κ, quantum efficiency of the detector.

*λ*, radiation wavelength.

ϕ, cylindrical angular coordinate in the diffraction space.

Φ, curve drawn by the end of normal vector **p** while the sample is rotated.

φ, phase of an electromagnetic wave.

ρ_e_, electron-density distribution in the sample.

Σ, scattering power of the sample.
